# Effects of Preharvest Application of Oxalic Acid, γ-Aminobutyric Acid, and Melatonin on the Microbiological and Physicochemical Quality of Dried Figs at Commercial Harvest and During Storage

**DOI:** 10.3390/toxins18030140

**Published:** 2026-03-13

**Authors:** Cristina Hidalgo, Santiago Ruiz-Moyano, Alicia Rodríguez, María G. Cordoba, Margarita López-Corrales, Manuel J. Serradilla

**Affiliations:** 1 Nutrición y Bromatología, Escuela de Ingenierías Agrarias, Universidad de Extremadura, 06007 Badajoz, Spain; 2 Instituto Universitario de Investigación en Recursos Agrarios (INURA), Avd. de la Investigación, Universidad de Extremadura, 06006 Badajoz, Spain; 3 Junta de Extremadura, Centro de Investigación Finca La Orden-Valdesequera (CICYTEX), Fruticultura, Autovía Madrid-Lisboa, s/n, 06187 Guadajira, Spain; 4 Área de Postcosecha, Valorización Vegetal y Nuevas Tecnologías, Centro de Investigaciones Científicas y Tecnológicas de Extremadura (CICYTEX), Instituto Tecnológico Agroalimentario de Extremadura (INTAEX), Junta de Extremadura, Avda. Adolfo Suárez, s/n, 06007 Badajoz, Spain

**Keywords:** elicitors, preharvest treatment, dried figs, physicochemical quality, fungal populations, mycotoxins, storage

## Abstract

The objective of this study was to evaluate the preharvest application of γ-aminobutyric acid (GABA), melatonin (MT), and oxalic acid (OA), at different concentrations and application frequencies, on the physicochemical and microbiological quality of dried figs (cv. Calabacita) at commercial harvest and after 3 and 6 months of refrigerated storage. A further aim was to determine their impact on fungal populations and mycotoxin production. The results showed that untreated dried figs had a higher frequency of *Aspergillus welwitschiae*, *A. tubingensis*, and *Aspergillus* section *Flavi*, whereas elicitor-treated figs exhibited a lower incidence of toxigenic fungi. *A. welwitschiae* was the main ochratoxin A (OTA)-associated species detected, although the proportion of OTA-positive figs was lower in elicitor-treated samples than in the control. Aflatoxins (AFs) were detected only sporadically in 2 mM OA treatments, consistent with the limited activity of *A. flavus* at low storage temperatures. Conversely, *Penicillium* spp. were widespread but were associated with citrinin (CIT) production only under 2 mM OA treatments. Among the *Alternaria* toxins, alternariol (AOH) was detected solely in dried figs treated with 1 mM OA. Notably, all investigated mycotoxins were below the limit of detection (<LOD) in dried figs treated with 0.5 mM MT. Moderate elicitor concentrations (e.g., 0.5 mM MT and 50 mM GABA) and multiple preharvest applications generally provided the best balance between fungal suppression and fruit quality, significantly reducing *Aspergillus* spp. occurrence without promoting the growth of undesirable species. Overall, elicitor treatments decreased the incidence of toxigenic fungi, most likely through direct antifungal effects in senescent dried fruit rather than by inducing host defences. The combined use of preharvest elicitors with appropriate drying and storage conditions is a promising strategy to control fungal contamination and mycotoxin accumulation in dried figs while maintaining quality from preharvest storage. Further research is needed to optimise elicitor concentrations and application timing.

## 1. Introduction

A notable increase in global fig production has been observed over the last two decades (2003–2023), particularly in harvested area, possibly due to new agricultural management practices and increased consumer demand. During this period, Spain has ranked among the world’s top ten producers, with an annual production of approximately 37,405.38 tonnes [1]. Extremadura is the main fig-producing region [2]. The ‘Calabacita’ variety is the predominant cultivar in Extremadura, owing to its high sensory quality [3].

In Extremadura, traditional fig cultivation for drying relies on sun-drying. As figs ripen and reach senescence at different times, they frequently fall to the ground, requiring several harvest rounds throughout the season [4]. The sun-drying process reduces moisture content to below 26%, typically achieved in farmers’ fields or greenhouses, in accordance with quality standard DDP-14 [5]. Although sun drying is the most environmentally friendly and cost-effective method, it has several field-related drawbacks. The slow drying rate, exposure to birds and pests, and variable environmental conditions lead to losses in nutritional and sensory quality, while also compromising microbiological safety by promoting conditions favourable to fungal growth and mycotoxin accumulation [6,7].

At an industrial scale, dried figs are fumigated with aluminium or magnesium phosphide (a gas called “phosphine”) to control insects. They are then sorted, briefly blanched, dried with hot air, checked for potentially dangerous fungal toxins (mycotoxins) under ultraviolet light, coated with flour, and packaged. Several storage steps, each lasting up to 6 months, are included. Throughout these stages, dried figs are exposed to changing temperatures and humidity for long periods, which promote fungal growth and mycotoxin production [8].

Fungal genera such as *Aspergillus*, *Alternaria*, *Penicillium*, and *Cladosporium* have frequently been reported in dried figs, from preharvest [9,10,11] through to storage [8,12,13]. Among these genera, several species can produce mycotoxins. Previous studies have reported the occurrence of aflatoxins (AFB_1_, AFB_2_, AFG_1_ and AFG_2_), ochratoxins A and B (OTA and OTB), fumonisin B_1_ (FB_1_), fusaric acid (FA), beauvericin (BEA), α-cyclopiazonic acid (CPA), patulin, mycophenolic acid (Myc Ac), alternariol (AOH), alternariol monomethyl ether (AME), tenuazonic acid (TeA), citrinin (CIT) and zearalenone (ZEA) in dried figs [7,8,14,15].

The European Union Commission (2023) set maximum levels (MLs) for mycotoxins in dried figs: 6.0 μg/kg for AFB_1_, 10.0 μg/kg for total AFs (sum of AFB_1_, AFB_2_, AFG_1_, and AFG_2_), and 8.0 μg/kg for OTA [16]. A proposed maximum for TeA is 1000 μg/kg, but none yet exist for AOH or AME [17].

Toxigenic fungi and mycotoxin synthesis in dried figs often start in the field. This occurs during fruit development, over-ripening, and senescence [9,10,14]. Prevention methods, such as the Good Agricultural Practices (GAP) from the World Health Organization (WHO) [18], can help mitigate these risks. Agronomic management and geographic location affect the occurrence of fungal species and mycotoxin contamination. Specifically, *Aspergillus* spp. is the predominant genus under rainfed conditions, with a high rate of aflatoxin-producing moulds [9,10]. Using meshes during fig harvesting reduces microbiological counts [19]. Additionally, fig-tree coverings and nets (Witty^®^ system) help control the levels of several mycotoxins [14].

Subsequently, several postharvest strategies have been developed to maintain the microbiological quality achieved during the preharvest period throughout prolonged storage, including the dehydration of fresh figs with osmotic or chemical pretreatments [20]; cold plasma treatment for *Aspergillus niger* inactivation in dried figs [21]; and antimicrobial coatings derived from *Ficus carica* latex [22].

Among current pre- and postharvest strategies, elicitors are increasingly being employed. Elicitors are natural compounds that, at low concentrations, trigger acquired or induced resistance in plants against biotic or abiotic stresses [23]. In vitro application of 0.001–1 mM melatonin (MT) has been shown to significantly inhibit spore germination of *A. flavus*, whereas in vivo tests revealed a reduction in *A. flavus* growth and AFB_1_ accumulation in fresh pistachio fruit treated with 1 mM MT [24]. Zengin et al. [25] applied MT (from 0.01 to 1 mM) to fresh apricot postharvest, prior to sun drying, to improve dehydration. Preharvest application of 20 and 40 mM oxalic acid (OA) reduced the severity of grey mould (*Botrytis* spp.) infections naturally occurring on stored strawberries [26]. Furthermore, postharvest application of γ-aminobutyric acid (GABA) has been shown to induce disease resistance in apples by regulating polyamine metabolism, the GABA shunt, and reactive oxygen species metabolism [27].

Despite the advantages of traditional sun drying, contamination with moulds and mycotoxins from preharvest to postharvest remains the main challenge in this sector. In this context, elicitors have demonstrated inhibitory effects on fungal growth and mycotoxin production in vitro. However, pre- or postharvest applications of elicitors have mainly focused on enhancing the nutritional and sensory quality of fruit, whereas in vivo studies have rarely evaluated the total spontaneous fungal population or the occurrence of mycotoxins in fruits. Therefore, the main objective of this study was to evaluate the effects of preharvest application of OA, GABA, and MT on the microbiological and physicochemical quality of dried figs at commercial harvest and after 3 and 6 months of cold storage, with particular emphasis on fungal diversity and mycotoxin profiles.

## 2. Results

### 2.1. Effect of Preharvest Elicitors on Physicochemical and Microbiological Quality of Dried Figs at Commercial Harvest

Preharvest elicitor applications significantly influenced the physicochemical parameters of dried figs (Table 1). Overall, moisture content, water activity (a_w_), total soluble solids (TSS), firmness, and skin colour (L* and C*) of dried figs were significantly affected by the harvest date and the number of applications. Moisture content ranged from 20.18 to 30.25% (Table 1), with higher values generally observed in the second harvest with two applications (H2_2app) and the first harvest with three applications (H1_3app), and lower values in the first harvest with two applications (H1_2 app; ~21%) and the second harvest with three applications (H2_3 app; ~23%). Regarding treatment effects, dried figs treated with 2 mM OA, 10 or 50 mM GABA, and 0.1 or 0.5 mM MT showed significantly lower moisture contents than the other treatments in specific harvest rounds. After two applications, although significant differences among treatments were detected, all elicitor-treated dried figs maintained moisture content comparable to that of the untreated control. Among figs subjected to three applications (H2_3 app), those treated with 50 mM GABA showed the lowest moisture content.

a_w_ values ranged from 0.448 to 0.573 (Table 1) and differed among treatments. Figs treated with C20, 10 mM GABA, and 1 mM OA had higher a_w_ values than the control in H1_2app and H1_3app; other treatments led to lower a_w_ values. After three applications, all elicitor-treated figs had significantly lower a_w_ values than untreated dried figs at the second harvest (H2_3app). TSS ranged from 79.23 to 85.00 °Brix (Table 1). Figs from H2_3app had the highest TSS, and those from H1_2app had the lowest. Except for 0.5 mM MT, H2_3app figs generally had higher TSS than the control. Overall, elicitor-treated figs had TSS similar to that of the control. The 0.1 mM MT treatment showed the highest TSS, the 10 mM GABA and 0.5 mM MT treatments had the lowest.

Dried fig firmness ranged from 0.66 to 2.19 N (Table 1). Differences were primarily associated with harvest time: figs from H2_3app had the highest firmness, whereas those from H2_2app and H1_3app had the lowest. For skin colour, L* values ranged from 50.84 to 63.77 (Table 1). Dried figs from H2_2app and H1_3app showed higher brightness (L*), whereas those from H1_2app had lower values. Considering the harvest and treatment interaction, all treated figs generally displayed L* values similar to the control within each harvest; however, in H2_3app, figs treated with 50 mM GABA and 0.1 mM MT showed significantly higher brightness than those treated with 2 mM OA (A2). C* values ranged from 33.44 to 39.74 (Table 1), with higher values recorded in dried figs subjected to three applications. In H2_3app, elicitor-treated figs generally exhibited C* values comparable to untreated fruit, except for those treated with 50 mM GABA and 0.1 mM MT, which showed higher chroma. Hue values ranged from 71.42 to 75.22 (Table 1), with no significant differences detected between treated and untreated dried figs.

The effect of preharvest elicitors on the microbiological quality of dried figs at commercial harvest is shown in Table S1. Mould and yeast counts ranged from 0.83 to 4.17 log CFU/g and from below the detection limit (<LOD) to 4.97 log CFU/g, respectively. Neither the number of applications, the type of elicitor, nor their interaction had a significant effect on fungal populations at this stage.

### 2.2. Effect of Preharvest Elicitor Treatments on the Physicochemical and Microbiological Quality of Dried Figs During Storage

Table S2 shows the effect of preharvest treatments on dried figs’ physicochemical quality after 3 and 6 months of cold storage. Storage time significantly affected moisture content, a_w_, TSS, firmness, and skin colour (L*, C*, and hue). Elicitor treatments and their interaction with storage time only affected TSS. Moisture content increased from harvest (24.34%) to 3 months (42.34%) and then decreased slightly at 6 months (40.73%). Similarly, a_w_ rose throughout storage, from 0.521 at harvest, to 0.699 at 3 months and 0.705 at 6 months. TSS decreased from 82.39 °Brix at harvest to 68.58 °Brix at 3 months and 66.49 °Brix at 6 months, with all treatments showing similar trends. Firmness fell sharply from 1.26 N at harvest to about 0.50 N after storage, mostly in the first 3 months. L* values ranged from 57.12 to 58.44 and changed little during storage (*p* = 0.044). C* values dropped from 36.71 to 32.51, showing reduced chroma; hue values rose from 73.67 to 76.23, indicating lighter tones. Table S3 shows that elicitor treatments significantly influenced mould counts (*p* = 0.038) and interacted with storage time for yeast populations (*p* = 0.029); storage time alone had no significant effects. Mould counts ranged from 3.01 to 3.11 log CFU/g, yeast from 0.99 to 1.18 log CFU/g. Significant treatment effects during storage but none observed at harvest suggests that elicitors act through preventive or induced resistance, rather than through direct fungicidal action.

### 2.3. Effect of Preharvest Elicitors on Fungal Diversity and Prevalence in Dried Figs at Harvest and After Storage

**Figure 1:**
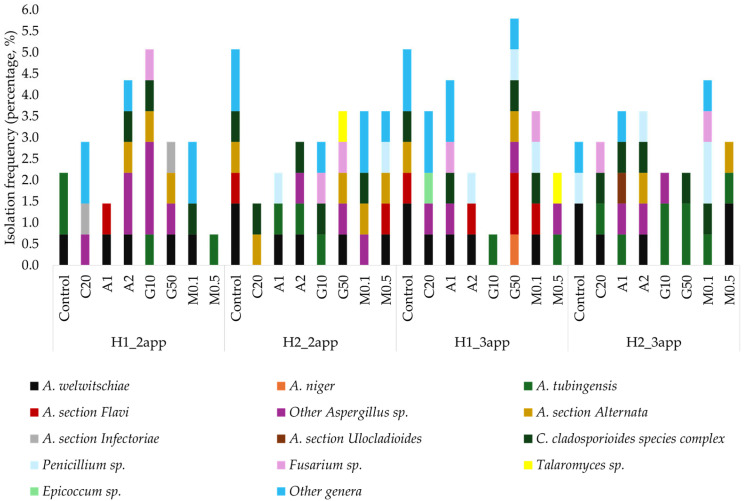
Isolation frequency (%) of mould species and genera from dried figs at commercial harvest. H1_2app: First harvest, two applications; H2_2app: Second harvest, two applications; H1_3app: First harvest, three applications; H2_3app: Second harvest, three applications; Control: untreated; C20: 0.2 mL/L Tween 20 treatment; A1: 1 mM OA treatment; A2: 2 mM OA treatment; G10: 10 mM GABA treatment; G50: 50 mM GABA treatment; M0.1: 0.1 mM MT treatment; M0.5: 0.5 mM MT treatment.

The distribution and frequency of fungal isolates across elicitor treatments and harvest times are shown in Figure 1. Specifically, at commercial harvest, a total of 138 fungal isolates were identified (Table S4), predominantly from *Aspergillus* (47.83%), a genus often associated with postharvest decay and mycotoxin production. *Cladosporium* (12.32%), and *Alternaria* spp. (10.87%) were also common, both for roles in spoilage and allergenic effects. The most frequent *Aspergillus* species were *A. welwitschiae* and *A. tubingensis*, with others, including *A. rugulosus*, *A. europaeus*, *A. uvarum*, *A. brasiliensis*, *A. terreus*, *A. melleus*, and *A. alliaceus*, also detected. Other isolated genera included *Botryosphaeria*, *Truncatella*, *Biscogniauxia*, *Stemphylium*, *Paecilomyces*, *Trichoderma*, *Albifimbria*, and *Boeremia*, all of which are frequently linked to plant diseases or spoilage.

Across harvests, the highest fungal isolation frequency occurred in H1_3app (27.54%). H2_2app (25.36%) and H2_3app (24.64%) followed, with H1_2app showing the lowest frequency (22.46%). Regarding treatments, the largest proportion of isolates was obtained from untreated dried figs (15.22%). Figs treated with 50 mM GABA and 0.1 mM MT followed. In H1_2app, figs treated with 0.5 mM MT and 1 mM OA showed lower fungal isolation frequencies (0.72–1.45%) than the control (2.17%). Other treatments exhibited higher fungal isolation frequency and diversity. In control figs, both *A. welwitschiae* (a potential OTA producer) and *A. tubingensis* were detected. These species were absent in figs treated with C20, 2 mM OA, 10 mM GABA, or 0.5 mM MT. *Aspergillus* section *Flavi* appeared only in 1 mM OA-treated figs. Some elicitors (2 mM OA, 10–50 mM GABA, and 0.1 mM MT) promoted greater fungal diversity, including *Alternaria*, *Cladosporium*, and *Fusarium* spp.

In H2_2app, all elicitor-treated dried figs showed lower fungal isolation frequencies (2.17–3.62%) than the untreated samples (5.07%). *A. welwitschiae* was mainly detected in controls. *A. tubingensis* appeared sporadically in figs treated with 1–2 mM OA and 10 mM GABA. *Aspergillus* section *Flavi* occurred only in control and 0.5 mM MT-treated dried figs. Other genera identified included *Cladosporium*, *Penicillium*, *Fusarium*, and *Talaromyces* spp.

In H1_3app, the highest fungal isolation frequency was recorded in dried figs treated with 50 mM GABA (5.80%). This was slightly higher than the control (5.07%). Other elicitors reduced fungal incidence (0.72–4.35%). *A. welwitschiae* was most frequent in untreated figs. *A. niger* appeared only in samples treated with 50 mM GABA. *A. tubingensis* was found in figs treated with 10 mM GABA and 0.5 mM MT. *Aspergillus* section *Flavi* was mainly detected in the 50 mM GABA treatment and at low levels in the control, 2 mM OA, and 0.1 mM MT treatments.

Finally, in H2_3app, figs treated with 10–50 mM GABA had the lowest fungal isolation frequencies (2.17%), compared with C20 and 0.5 mM MT (2.90%) or higher levels under other treatments (3.62–4.35%). *A. welwitschiae* was detected primarily in controls and 0.5 mM MT-treated figs, while *A. tubingensis* was detected in most treatments, except 2 mM OA and the control. Other *Aspergillus* spp., together with *Alternaria*, *Cladosporium*, *Penicillium*, *Fusarium*, *Talaromyces*, and *Epicoccum* spp., were also isolated.

**Figure 2:**
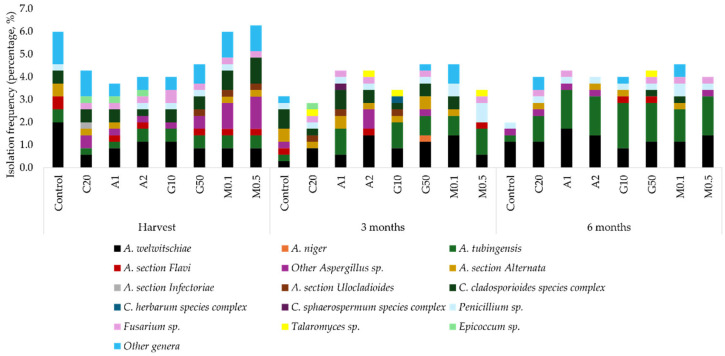
Isolation frequency (%) of mould species and genera from dried figs during storage (harvest, 3 months, and 6 months). Control: untreated; C20: treated with 0.2 mL/L Tween 20; A1: treated with 1 mM OA; A2: treated with 2 mM OA; G10: treated with 10 mM GABA; G50: treated with 50 mM GABA; M0.1: treated with 0.1 mM MT; M0.5: treated with 0.5 mM MT.

During storage, the composition and prevalence of fungal isolates changed markedly (Figure 2). A total of 352 fungal isolates were identified, predominantly belonging to *Aspergillus* (57.39%), *Cladosporium* (10.23%), and *Alternaria* spp. (7.67%). Overall, total fungal isolation frequency in dried figs declined from 38.74% at harvest to 30.48% after 3 months and 31.05% after 6 months of storage. Across treatments, elicitor-treated dried figs showed higher total fungal isolation frequencies (11.40–15.10%) than untreated or C20-treated figs; however, this metric encompasses both toxigenic and non-toxigenic isolates. The specific effects of treatment on toxigenic fungi are addressed separately below at the genus- and species-levels.

*A. welwitschiae* was the dominant species (23.36%), detected under all conditions. At harvest, its frequency was higher in untreated figs (1.99%) than in treated ones (0.85–1.14%). After 3 months, it was more common in treated dried figs (0.57–1.42%) than in the control (0.28%). After 6 months, similar frequencies (1.14%) were found in untreated, C20-, 50 mM GABA- and 0.1 mM MT-treated dried figs. Figs treated with 1–2 mM OA and 0.5 mM MT had higher values (1.42–1.71%). The lowest frequency (0.85%) appeared in 10 mM GABA-treated figs.

*A. tubingensis* was the second-most prevalent species. At harvest, C20- and 1 mM OA-treated figs had lower frequencies (0.28%) than the control and other treatments (0.57%). Over the next 3 months, *A. tubingensis* remained undetected in C20- and 2 mM OA-treated dried figs, while in other treatments, its frequency increased to 0.85–1.14%, exceeding the control. By 6 months, all elicitor-treated dried figs (1.42–1.99%) had higher *A. tubingensis* frequencies than untreated samples (0.28%), indicating a reversal of the earlier trend and a stronger increase from earlier timepoints compared to the control.

*A. niger* appeared only in 50 mM GABA-treated figs after 3 months. At harvest, *Aspergillus* section *Flavi* was more frequently isolated from untreated dried figs (0.57%) than from OA-, 50 mM GABA- and MT-treated samples, where it was detected at lower rates. During storage, detection of this section in 2 mM OA- and 0.5 mM MT-treated figs after 3 months, and in GABA-treated figs only after 6 months, indicating delayed or reduced occurrence compared to untreated figs.

### 2.4. Influence of Preharvest Elicitors on Mycotoxin Profiles of Dried Figs During Storage

Table 2 summarises the occurrence of mycotoxins detected in dried figs treated with elicitors after six months of storage. Of the 16 mycotoxins analysed, six were detected at varying concentrations (Table 2). Overall, ochratoxin A (OTA) was the most prevalent, followed by ochratoxin B (OTB), aflatoxin B_1_ (AFB_1_), aflatoxin B_2_ (AFB_2_), citrinin (CIT), and alternariol (AOH). The remaining compounds—α-cyclopiazonic acid (CPA), mycophenolic acid (Myc Ac), zearalenone (ZEA), alternariol monomethyl ether (AME), sterigmatocystin (STG), o-methylsterigmatocystin (OM-STG), griseofulvin (GRIS), and fumonisin B_1_ (FB_1_)—were below the limit of detection (<LOD). OTA was detected in all treatments except M0.5 (100% <LOD), with no samples exceeding the legal limit (LL). Compared with the control (33.3% in the LOD-LL range), all elicitor treatments showed a lower incidence of samples within the LOD-LL range (25%), whereas C20 exhibited a higher incidence (50%). OTB levels were <LOD in all cases except for G50, where 25% of samples fell within the LOD-LL range, coinciding with OTA occurrence. AFB_1_, AFB_2_, and CIT were <LOD in all treatments except A2, where 25% of samples contained AFB_1_ (LOD-LL), AFB_2_ (>LL), and CIT (>LL). AOH was <LOD in all treatments except A1, where 25% of samples were within the LOD-LL range.

### 2.5. Correlation of Variables

**Figure 3:**
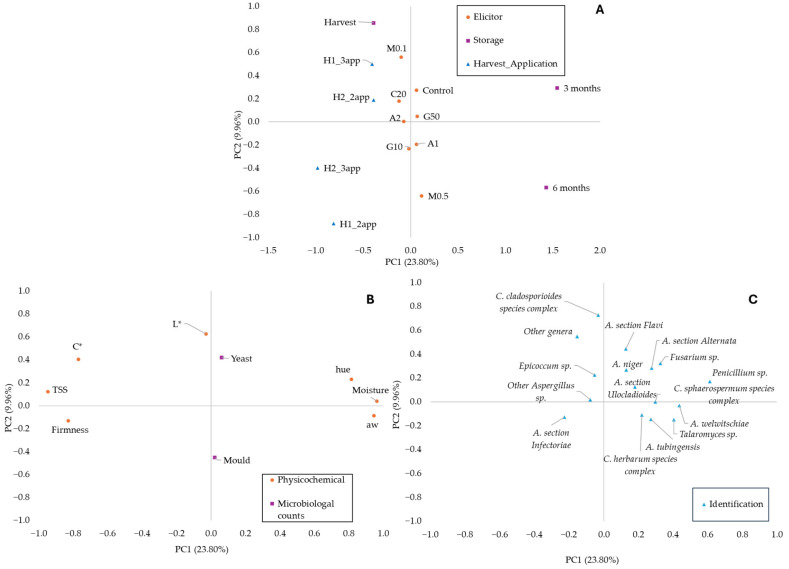
Principal component analysis (PCA). (**A**) Treatments (circles), storage (squares), harvest x application schedule (triangles). (**B**) Physicochemical parameters (circles), microbiological counts (squares). (**C**) Fungal identifications (triangles). Moisture; a_w_: water activity; TSS: total soluble solids; L*: brightness; C*: chroma; hue; mould and yeast counts; *A. welwitschiae; A. niger, A. tubingensis*; *A*. section *Flavi*; Other *Aspergillus* spp.; *Alternata* (*Alternaria* section *Alternata*)*; Infectoriae* (*Alternaria* section *Infectoriae*); *Ulocladioides* (*Alternaria* section *Ulocladioides*); *Herbarum* (*Cladosporium herbarum* species complex); *Cladosporioides* (*Cladosporium cladosporioides* species complex), *Sphaerospermum* (*Cladosporium sphaerospermum* species complex); *Penicillium* spp.; *Fusarium* spp.; *Talaromyces* spp.; *Epicoccum* spp., other genera. H1_2app: First harvest, two applications; H2_2app: Second harvest, two applications; H1_3app: First harvest, three applications; H2_3app: Second harvest, three applications; Control: untreated; C20: 0.2 mL/L Tween 20; A1: 1 mM OA; A2: 2 mM OA; G10: 10 mM GABA; G50: 50 mM GABA; M0.1: 0.1 mM MT; M0.5: 0.5 mM MT.

PCA was performed (Figure 3) to assess the impact of preharvest elicitor applications on the physicochemical and microbiological quality, and fungal population of dried figs during storage. The first two principal components explained 33.76% of the total variance (23.80% and 9.96%, respectively). Dried figs from H2_2app and H1_3 app were positioned in the negative scores of PC1 and the positive scores of PC2. These figs were associated with higher TSS, colour parameters (L* and C*), and the presence of *C. cladosporoides* species complex, *Epicoccum* spp., and other *Aspergillus* spp. In contrast, H1_2app and H2_3app showed negative scores in both PCs. They correlated with greater firmness and the detection of *Alternaria* section *Infectoriae*. Untreated figs and those treated with C20, 1–2 mM OA, and 10–50 mM GABA clustered near the centre of the PCA plot. In comparison, 0.1 mM and 0.5 mM MT treatments were clearly separated, indicating distinct effects. Regarding storage time, dried figs at commercial harvest were located in the negative scores of PC1 and the positive scores of PC2. Figs stored for six months appeared on the opposite side, associated with higher a_w_, mould counts, and the presence of *A. welwitschiae*, *A. tubingensis*, *C. herbarum* species complex, and *Talaromyces* spp. Dried figs stored for three months occupied the positive scores of both PCs and were linked to higher moisture content, hue values, yeast counts, and the presence of *Aspergillus* section *Flavi*, *A. niger*, *A.* section *Alternata*, *A.* section *Ulocladioides*, *C. sphaerospermum* species complex, *Fusarium*, and *Penicillium* spp. Overall, the PCA indicated that dried figs at commercial harvest differed slightly from those stored for three months. However, they were clearly distinct from figs stored for six months.

## 3. Discussion

Dried figs in Extremadura are traditionally produced by sun-drying, yielding fruit with excellent sensory and nutritional qualities. However, this natural drying process also exposes the fruit to environmental factors that promote fungal contamination and subsequent mycotoxin accumulation [28]. The microbiological safety of traditionally sun-dried figs could be enhanced through natural strategies that help preserve their intrinsic characteristics. In other crops, preharvest applications of elicitors or biostimulants have been shown to enhance physicochemical and nutritional properties, while in vitro studies have demonstrated their ability to reduce fungal growth and mycotoxin accumulation [23]. Therefore, in this study, two and three preharvest applications of OA, MT, and GABA were applied to fig trees by foliar spraying. The effects of these elicitors on the physicochemical and microbiological quality of the resulting dried figs were then evaluated at commercial harvest and after storage.

In this study, the physicochemical traits of dried figs at harvest were consistent with those reported by Galván et al. [28] and Galán et al. [14] for ‘Calabacita’ dried figs produced in Extremadura. In contrast, Arroul et al. [29] reported similar moisture content but higher a_w_ values in Algerian dried figs compared with those obtained in H1_2app. Moreover, the physicochemical characteristics of dried figs at commercial harvest were influenced by both the timing and frequency of elicitor applications. These differences may be attributed to variations in ambient temperature during the harvesting weeks, which directly affect fruit moisture at harvest and during subsequent drying. Indeed, moisture content was highest in H2_2app and H1_3app (harvested on the same date), mirroring the mean drying temperatures recorded: 23.8 °C during H2_2app/H1_3app compared with 31.1 °C in H1_2app and 30.2 °C in H2_3app (Figure S1). These temperature-driven differences in moisture loss during drying likely explain the observed variations in TSS and firmness among harvesting times.

Toxigenic fungal contamination and mycotoxin occurrence remain among the most critical issues affecting the production of dried figs [28]. In this context, moisture content and a_w_ are key parameters for their control. During the drying process, both moisture and a_w_ decrease, while desirable sensory attributes develop [14]. In fresh fruit, elicitor application has been shown to mitigate moisture loss [30]. However, in dried fruits, achieving the target dehydration level as efficiently as possible is of particular importance. Zengin et al. [25] reported that postharvest application of MT (0.01, 0.1, and 1 mM) to fresh apricots improved the subsequent sun-drying process.

A yellow skin colour in dried figs is considered desirable, and its preservation is influenced by several pre- and post-harvest factors. Colour development is strongly affected by environmental conditions and light intensity during fruit development and senescence [19]. High solar radiation may explain the variations observed in skin colour parameters (L* and C*) between harvest times. Dried figs subjected to three applications exhibited higher C* values than those with two applications. A higher chroma value indicates greater suitability for the drying process [25], ultimately resulting in a brighter colour that can enhance consumer acceptance [31]. In this study, elicitors helped preserve desirable colour characteristics of dried figs at harvest. In particular, H2_3app samples treated with 50 mM GABA or 0.1 mM MT showed the highest L* and C* values. Similarly, Zengin et al. [25] found that a postharvest treatment with 0.1 mM MT improved colour parameters in sun-dried apricots, suggesting that melatonin can prevent darkening and enhance colour brightness. Furthermore, Badiche et al. [32] reported that the citrus colour index of lemons increased proportionally with higher preharvest GABA concentrations, supporting the colour-enhancing potential of this compound.

A high TSS content and elevated sugar levels are desirable quality attributes in dried figs [33]. In this study, TSS values varied with harvest timing and with both the type and concentration of the elicitor, as observed in earlier studies. Zengin et al. [25] found that postharvest application of 0.1 mM MT enhanced sugar content in sun-dried apricots. Preharvest applications of 1 and 3 mM OA increased strawberry TSS, while 2 mM OA maintained comparable levels [30]. This pattern emphasises the importance of concentration in elicitor performance. Conversely, Badiche et al. [32] reported that preharvest GABA at 10, 50, or 100 mM did not significantly affect TSS in lemon fruits across two seasons or two ripening stages (green and yellow). Taken together, these studies suggest that harvest timing and drying conditions, in combination with elicitor treatment, can significantly influence TSS outcomes. Notably, H2_3app figs exhibited both higher TSS and firmness. This is significant, as firmness usually decreases during ripening and senescence. Therefore, three preharvest applications, especially of GABA and MT, may delay senescence or enhance cell wall integrity, preserving fruit texture even at advanced maturity.

In our study, elicitors did not adversely affect the firmness of dried figs at harvest. To accurately assess the influence of elicitors on fruit firmness, it is essential to evaluate a range of concentrations. For example, Anwar et al. [30] demonstrated that preharvest applications of 1 and 2 mM OA increased strawberry firmness at harvest, whereas 3 mM OA maintained texture levels.

Traditional processing of dried figs involves prolonged storage [8] during which it is essential to preserve the physicochemical quality achieved at harvest. Therefore, the effects of preharvest applications of OA, GABA, and MT were evaluated after 3 and 6 months of cold storage (8 °C). Moisture content and a_w_ of dried figs increased during storage, exceeding the recommended sanitary safety threshold of 26% moisture [5]. These findings are consistent with previous reports. For example, ‘Calabacita’ sun-dried figs stored at 8 °C after blanching showed increases in moisture content and a_w_ of up to 30% and 0.70, respectively [8]. Similarly, Villalobos et al. [20] reported that moisture levels rose from 22.22% to 25.54% in dried figs stored at 20 °C and 65–75% RH for 90 days.

TSS content in dried figs decreased significantly during storage, as also reported by Villalobos et al. [20]. In their study, the TSS of ‘Calabacita’ sun-dried figs stored at 20 °C and at 65–75% RH remained stable until day 70, before declining by day 90, within a range of 37.67–37.99 °Brix. In our study, differences among treatments were also observed throughout storage. Likewise, in other fruit, Fekry et al. [34] reported that preharvest application of MT (0.043–0.215 mM) increased the TSS of fresh date palm fruits stored at 4 °C for 28 days.

In line with the observed increase in moisture content, fruit firmness decreased during storage. Fortunately, a soft texture is considered a desirable attribute in dried figs, contributing positively to consumer acceptance [35]. However, under controlled relative humidity conditions (65–75% RH) for 90 days, the firmness of ‘Calabacita’ sun-dried figs was reported to increase from approximately 1 to 2 N [20]. Similarly, in fresh date palm fruits stored at 4 °C, preharvest application of MT enhanced firmness [34]. Dried figs maintained their luminosity from harvest through storage, while C* values decreased and hue angle increased. Preserving the characteristic bright yellow skin colour of dried figs is essential for consumer acceptance [31]; therefore, postharvest handling should aim to minimise colour alterations [19]. Ansari et al. [35] reported that increased moisture levels promote browning reactions, making darkening one of the most critical challenges during the storage of dried figs. Lower C* values reflect reduced colour vividness and overall quality, likely due to storage effects.

The mould and yeast counts of dried figs were not significantly affected by harvest timing, elicitor type or concentration, or storage duration. The counts found at harvest matched those for ‘Calabacita’ dried figs from Extremadura, as reported by Galán et al. [14], with yeast and mould counts ranging between 2.1–5.3 log CFU/g and 2.2–3.1 log CFU/g, respectively. Because dried figs are often harvested from the ground multiple times each season, microbial counts tend to remain stable regardless of harvest timing. However, the fruit’s physiological state and environmental conditions at harvest could influence fungal populations. Notably, even though moisture content and a_w_ content increased during storage, mould counts remained steady. Galván et al. [8] also reported stable yeast and mould counts, from 2.16 to 3.13 and 2.00 to 3.26 log CFU/g, respectively, with no significant differences between curing and after storage. In contrast, Villalobos et al. [20] observed that after 90 days of storage, an increase in moisture was associated with a decrease in mould counts to <LOD.

Although mould counts were similar across treatments in our study, the primary concern in dried figs is the occurrence of OTA and AFs, produced by *Aspergillus* section *Nigri* and *Aspergillus* section *Flavi*, respectively. Consequently, the most critical aspect of the microbiological evaluation is the characterisation of fungal population composition in dried figs, taking into account the effects of harvest timing, preharvest elicitor treatments, and storage duration. Moreover, given that the expected commercial shelf life of dried figs is six months, mycotoxins were assessed only at the end of storage (6 months); consequently, intermediate storage times were not evaluated for these compounds. Currently, the occurrence of toxigenic fungi in stored-dried figs remains a critical concern [36]. *Aspergillus* spp., *Alternaria* spp., and *Penicillium* spp. have been widely reported in field-grown dried figs [9,10,11] and during storage [8,12,13]. Specifically, at harvest in ‘Calabacita’ dried figs, Galán et al. [9] found that the majority of the fungal population belonged to *Aspergillus* spp. (69%), with 24.5% *A. welwitschiae*, 21.5% *A. tubingensis*, 6.5% *A. niger* and 6% *A. flavus*. Although the same genera and species were detected in our study, Galván et al. [10] reported a different distribution, with *Penicillium* spp. (29.4%) being the most frequent, followed by *Aspergillus* spp. (24.5%) and *Alternaria* spp. (17.3%), with individual *Aspergillus* species representing 6.2% *A. flavus*, 5.9% *A. tubingensis*, and 4.5% *A. welwitschiae*.

Other authors suggest drying at temperatures above 30 °C, ideally near 37 °C, to control *A. flavus* growth [37]. In H2_3app, the mean temperature was 31 °C, and the maximum was 41.37 °C; *A. flavus* was not detected. In H1_2app, daily temperature changes may have promoted *A. flavus* growth despite higher temperatures. Differences in fungal isolation frequency by harvest time may relate to environmental temperature and its effect on moisture. Higher drying temperatures speed dehydration and reduce moisture, lowering fungal isolates. Training trees on espaliers (two-dimensional planes) allows more uniform fig drying, and white weed-control mesh helps reduce fungal contamination.

Overall, untreated dried figs exhibited a higher frequency of *A. welwitschiae*, *A. tubingensis*, and *Aspergillus* section *Flavi* compared with figs treated with elicitors. Notably, most *Aspergillus* species were not detected following the application of certain elicitors. During postharvest processing of ‘Calabacita’ dried figs, *A. tubingensis* was the dominant species, followed by *A. welwitschiae* and *A. flavus* [8]. However, this pattern changed during long-term storage: while moisture content increased, fungal isolation frequency decreased, likely due to refrigeration (<10 °C).

OTA could be produced by *A. welwitschiae*, as this species was detected in both treated and untreated samples. *A. niger* was only detected in 50 mM GABA-treated figs after 3 months, while *A. tubingensis* is not an OTA producer [38]. Dried figs treated with 0.5 mM MT showed OTA levels below the limit of detection (<LOD), even though *A. welwitschiae* was present. Nevertheless, the occurrence of a mould does not necessarily imply the presence of its associated mycotoxin, and vice versa. In the Spanish industry, Galván et al. [8] detected OTA at certain processing stages, but not after storage. Similarly, Şenyuva et al. (2008) detected OTA in 32 of 50 dried fig samples removed from industrial processing lines [39]. At the commercial level, Wang et al. [40] reported no detectable OTA in retail samples from China, whereas Celik and Kabak [41] found OTA contamination (0.15–1.72 µg/kg) in eight dried fig samples collected from Ankara, Çorum, Istanbul, and Izmir (Turkey).

*Aspergillus* section *Flavi* was detected only sporadically during storage. This limited detection aligns with Galván et al. [37], who recommended maintaining refrigeration temperatures below 10 °C during industry processing, storage, and retailing of dried figs to prevent *A. flavus* growth and AFs production. Such conditions could explain the low frequency of AF detection. In A2 dried figs, AFs were detected after six months, while *Aspergillus* section *Flavi* was present at harvest and after three months, indicating that mycotoxin production occurred before the third month of storage. Furthermore, in the Spanish industry, Galván et al. [8] reported AFs at several processing stages (curing, blanching, and storage: 12.5–50% of samples, 0–75 µg/kg AFB_1_, and 0–22 µg/kg AFB_2_). However, only 12.5% of final products contained aflatoxins (50–75 µg/kg AFB_1_; 12–22 µg/kg AFB_2_). In contrast, Azaiez et al. [42] found no detectable levels of AFB_1_, AFB_2_, AFG_1,_ or AFG_2_ in Spanish trade samples. Similarly, in different types of retail trade, Celik and Kabak [41] detected aflatoxin contamination in 14 samples with AFB_1_ (0.26–11.92 µg/kg), 7 with AFB_2_ (0.10–0.68 µg/kg), and 2 with AFGs, whereas Wang et al. [40] reported 3 samples containing AFB_1_ (1.8–384.1 µg/kg), 1 with AFB_2_ (2.4–2.6 µg/kg), 3 with AFG_1_ (0.4–17.8 µg/kg) and 3 with AFG_2_ (0.6–1.2 µg/kg).

Species within *Aspergillus* section *Flavi* are also known producers of α-cyclopiazonic acid (CPA), and O-methyl sterigmatocystin (OM-STG) [43]. *Penicillium* spp. are producers of OTA, CPA, CIT, PAT, Myc Ac, and GRIS [44]. Although *Penicillium* spp. were isolated from both treated and untreated samples after 6 months, CIT was detected only in A2, where *Aspergillus* spp. were also present, suggesting these species as potential producers [44,45]. *Alternaria* toxins without current regulatory limits—AOH, AME, tentoxin (TEN), and altenuene (ALT)—were not detected by López et al. [46] in dried figs from retail outlets in the Netherlands, whereas AME and AOH were reported by Galán et al. [14] in the same area as our study (Guadajira, Extremadura) and by Wang et al. [40] in Chinese trade samples. By contrast, tenuazonic acid (TeA) was detected by López et al. [46] and Sulyok et al. [15] but was reported below the limit of detection (<LOD) by Galán et al. [14]. Other mycotoxins analysed were not detected in this study. In retail and trade samples, fumonisins (FB) and zearalenone (ZEA) were below detectable levels in dried figs from Spain [42] and China [40], respectively.

Previous research has demonstrated the decontamination potential of elicitors in fresh fruit and vegetables; however, little is known about their effectiveness in dried fruit. It should be noted that dried figs are senescent products with largely inactive metabolism. Therefore, the effects of elicitors on toxigenic moulds in dried figs are likely to result from direct antifungal activity rather than from the stimulation of the fruit’s defence mechanisms, as might occur in fresh figs. To illustrate these differences, Embaby et al. [47] reported a reduction of *A. parasiticus* isolated from fresh figs following in vitro treatment with ascorbic acid and benzoic acid. In support of the role of defence mechanisms in fresh produce, in vivo applications of elicitors such as GABA and OA have been shown to enhance defence responses in apple and kiwifruit, respectively [27,48]. Specifically, postharvest application of 0.5, 1, and 2 mM GABA induced resistance against *P. expansum* in apples [27]. Additionally, in vitro treatment with 0.001–1 mM MT significantly inhibited *A. flavus* spore germination, whereas in vivo application of 1 mM MT reduced both *A. flavus* growth and AFB_1_ accumulation in fresh pistachio fruits [24]. Further expanding the evidence, Petrić et al. [49] applied postharvest treatments with 0.5% citric acid, 0.5%ascorbic acid or 0.3% L–cysteine solution to fresh figs intended for artificial drying, finding that OTA remained below detectable levels, AFB_1_ was inhibited or reduced, and AME levels were decreased by L-cysteine and citric acid. Conversely, other mycotoxins—ochratoxin α (OTα), anthraquinone (ATN), emodin (EMO), tryptophol (TryOH), and brevianamide F (BREF)—were variably affected, being either reduced or enhanced depending on the treatment [49].

## 4. Conclusions

Preharvest application of elicitors modified fungal populations and mycotoxin profiles in dried figs while maintaining overall fruit quality from harvest through storage. Untreated figs showed a higher incidence of *Aspergillus welwitschiae*, *A. tubingensis*, and *Aspergillus* section *Flavi* at harvest, although refrigerated storage (8 °C) reduced fungal isolation frequency and mycotoxin production. *A. welwitschiae* was the main OTA-related species detected; however, the proportion of OTA-positive samples was lower in elicitor-treated figs than in controls. Aflatoxins were detected only sporadically under 2 mM OA treatments, *Penicillium* spp. were widespread but associated with CIT production only at 2 mM OA, and AOH was detected exclusively in figs treated with 1 mM OA. Notably, all mycotoxins analysed were below the limit of detection in figs treated with 0.5 mM MT. Moderate elicitor concentrations (e.g., 0.5 mM MT and 50 mM GABA) and multiple preharvest applications generally offered the best compromise between fungal suppression and fruit quality, reducing *Aspergillus* spp. occurrence without favouring undesirable species. In contrast, excessive or insufficient doses tended to increase fungal variability or reduce efficacy. Overall, elicitor treatments reduced the incidence of toxigenic fungi, most likely through direct antifungal effects in the senescent dried fruit. The combined use of elicitors with appropriate drying and refrigerated storage emerges as a promising strategy to significantly reduce fungal contamination and mycotoxin accumulation in dried figs. Further research is needed to refine optimal concentrations and application timing under commercial conditions.

## 5. Materials and Methods

### 5.1. Plant Material and Sampling

#### 5.1.1. Plant Material and Experimental Design

The fig orchard (cv. ‘Calabacita’) was located at the Centre for Scientific and Technological Research of Extremadura (CICYTEX) (Finca La Orden-Valdesequera; latitude 38° 85′ 80″ N, longitude −6° 66′54″ W; Guadajira, Badajoz, Spain), at an altitude of 217 m above sea level. The trees were planted in 2019 in an espalier formation within a super-intensive system (3 × 2.5 m spacing) and managed according to standard agronomic practices. The experiment used a randomised block design with three blocks. Each treatment comprised two or three applications, with six single-tree biological replicates per block, for a total of 18 trees per treatment. One tree served as the experimental unit. In total, 270 trees were used in the study. To prevent bird damage, the orchard was covered with an anti-bird net, and the soil surface was protected with an anti-weed mesh to minimise contamination from soil-borne fungal spores. During the experimental season (July and August 2023), no precipitation (0 mm) was recorded at Finca La Orden according to REDAREX [50], while daily temperature and relative humidity fluctuations are shown in Figure S1.

#### 5.1.2. Preharvest Application of Elicitors

Elicitor solutions were prepared in water at two concentrations for each compound: GABA at 10 and 50 mM, OA at 1 and 2 mM, and MT at 0.1 and 0.5 mM. These concentrations were selected based on previous studies of preharvest applications of GABA [51], OA [52], and MT [53] in pomegranate, fresh fig, and plum, respectively. Tween 20 (0.2 mL/L; *v*/*v*) was added as a surfactant to all solutions. For treatment, trees were divided into groups that received specific compounds at specific concentrations and application regimes. Foliar sprays were applied at dusk, using 1.2 L of freshly prepared solution per tree. The first spray was applied when fruits reached physiological maturity (30–35 mm in diameter), with one or two additional applications at one-week intervals, depending on the treatment, for a total of 2 or 3 applications per regime. Each compound/concentration/regime combination involved 18 trees (6 per block), with each tree serving as the experimental unit. For each application regime (two or three), eighteen trees treated with only water and Tween 20 served as the surfactant control (C20), and an additional set of eighteen fig trees remained untreated as a negative control (Table 3).

#### 5.1.3. Sampling During Commercial Harvesting of Dried Figs

Following these commercial harvests, the post-harvest handling of the fruits was consistent: after manual collection at two time points (based on elicitor applications, see Table 3), figs were dried under natural solar conditions. Analytical procedures were then performed at the commercial harvest stage after solar drying (Table 3).

#### 5.1.4. Storage Conditions of Dried Figs

To assess post-harvest quality dynamics, figs from the commercial harvests (from both elicitor treatments) were stored in bulk boxes at 8 °C and 70% relative humidity for 6 months in the dark, thereby simulating industry storage conditions. Sampling occurred after 3 and 6 months (Table 3).

### 5.2. Quality Parameters

#### 5.2.1. Physicochemical Parameters of Dried Figs

From each sampling point, 60 undamaged dried figs were selected for firmness and skin colour analysis. This selection enabled comparison of quality indicators across different treatments and time points. Firmness and skin colour were measured as described by Galván et al. [28]. The fruit was then homogenised to determine moisture content, water activity (a_w_), and total soluble solids (TSS). Moisture content was determined according to AOAC [54], and a_w_ was measured using a LabMASTER-a_w_ neo device (Novasina AG, Lachen, Switzerland).

#### 5.2.2. Mould and Yeast Counts

For microbial testing, 10 g of dried fig sample (per replicate, *n* = 3) were homogenised in 90 mL of sterile 0.1% (*w*/*v*) peptone water using a Stomacher (Lab Blender, Model 4001, Seward Medical, London, UK). Selecting three replicate samples per condition improved reliability and captured sample variability. Subsequently, tenfold serial dilutions were prepared in peptone water, and 0.1 mL aliquots were spread onto Potato Dextrose Agar (PDA, Scharlab, Barcelona, Spain) acidified to pH 3.5 with 10% (*w*/*v*) tartaric acid. Mould and yeast counts were determined after incubation at 25 °C for 5 days. Results were expressed as log CFU/g.

#### 5.2.3. Isolation and Identification of Moulds from Dried Figs

##### Isolation

One isolate of each morphotype was selected from the highest dilution of the PDA plates. Morphotypes were defined based on macroscopic colony characteristics, considering visual features such as the colour and overall appearance/shape of the mould mycelium [55]. The isolates were subcultured onto fresh PDA plates to obtain pure colonies. After incubation for 5 days at 25 °C, the mycelium was collected into a sterile Eppendorf vial for subsequent identification and stored at −20 °C. In addition, spores were harvested using 5 mL of sterile distilled water containing 0.05% (*v*/*v*) Tween 80 and stored in glycerol at −80 °C.

##### DNA Extraction

DNA extraction from fungal mycelium was performed using the Quick-DNATM Fungal/Bacterial Miniprep Kit (Zymo Research, CA, USA), following the manufacturer’s instructions. DNA concentration and purity were measured using a Nanodrop^®^ ND-1000 spectrophotometer (Thermo Fisher Scientific, Waltham, MA, USA).

##### Molecular Identification of Fungal Isolates

To identify fungal isolates at the species level, several genes were amplified and sequenced. For all isolates, partial β-tubulin (*Bt*) gene sequences were obtained using the primers Bt2a and Bt2b, as described by Glass and Donaldson [56]. Species identification was confirmed based on the following criteria: *Aspergillus niger* and *A. welwitschiae* were differentiated by comparing specific nucleotide positions [57]. Isolates with TA were identified as *A. welwitschiae*; those with GT as *A. niger*; and those with GA were further confirmed by sequencing the calmodulin (*CaM*) gene [58]. Other *Aspergillus* species were confirmed by *CaM* gene sequencing [58]. *Alternaria* species were confirmed by amplification of the glyceraldehyde-3-phosphate dehydrogenase (*gpd*) gene [59]. Other fungal genera (e.g., *Cladosporium* spp., *Penicillium* spp., *Fusarium* spp.) were confirmed using the internal transcribed spacer (ITS1/ITS2-5.8 S rDNA) region [60]. PCR reactions were performed in an Eppendorf Mastercycler^®^ nexus gradient (Madrid, Spain) under the conditions described by Gallardo et al. [61] for *Bt*, Perrone et al. [58] for *CaM*, by Woudenberg et al. [62] for *gpd*, and Galán et al. [9] for ITS. Amplification products were separated by electrophoresis on 1% agarose gels, and stained with Midori Green Advance (Nippon, Japan). PCR products were purified using the NucleoSpin Gel and PCR Clean-up kit (Macherey-Nagel GmbH & Co. KG, Düren, Germany) and sequenced by the Service of Bioscience Applied Techniques (STAB), Universidad de Extremadura (Badajoz, Spain). The GenBank accession numbers for the sequences used for species identification are provided in Table S4.

##### Sequence Analysis

Sequences were edited with BioEdit version 7.2 and checked against the NCBI database using BLAST (version +2.17.0). Species were identified based on the highest similarity (over 96%).

#### 5.2.4. Mycotoxin Extraction and Analysis

Following the editing and identification of sequences, mycotoxin identification and quantification were performed at the end of the storage period to simulate the complete process from dried fig harvest to maximum storage under commercial conditions. Mycotoxins were extracted from a homogenate of 60 dried figs using acetonitrile/water/acetic acid (79:20:1, *v*/*v*/*v*) at a ratio of 4 mL per gram of sample [15]. The mixtures were incubated for 90 min under continuous agitation and centrifuged at 4000 rpm for 10 min. The extracts were diluted 1:1 (*v*/*v*) with acetonitrile/water/acetic acid (20:79:1, *v*/*v*/*v*), filtered through a 0.22 µm nylon membrane, and stored at −80 °C. Multimycotoxin analysis was performed using a Q Exactive Plus mass spectrometer (Thermo Fisher Scientific, Waltham, MA, USA), following the procedure developed by Cebrián et al. [63], with modifications reported by Galán et al. [14] (Table S5).

#### 5.2.5. Statistical Analysis

Statistical analyses were performed using IBM SPSS Statistics 21 (IBM Corp., Armonk, NY, USA). We measured physical and chemical characteristics and the number of microorganisms, and analysed the results to see if there were differences using a general linear model for repeated measurements. Tukey’s and Bonferroni corrections were used to compare specific groups (*p* < 0.05). For the commercial harvest, the main factor within each group was the harvest time after two or three applications, and the main factor between groups was the treatment: chemical inducers at two levels, no treatment, or Tween 20. For storage, the within-group factor was storage time, and the between-group factor was again the treatment. We used averages and standard deviations to help understand the data.

Fungal incidence was expressed as total isolation frequency (%) at commercial harvest and during storage (Equation (1)). For each fungal type, isolation frequency was calculated for each treatment across all harvest/application dates and at each storage sampling point (harvest, 3, and 6 months).

(1) Total isolation frequency (%) = (Number of times a fungus was found/Total number of fungi found at that time) × 100

Mycotoxin occurrence at six months of storage was expressed as frequency (%) (Equation (2)) within three concentration ranges: below the detection limit (<LOD), between the detection limit and the legal limit (LOD–LL), and above the legal limit (>LL). The LL was set at 8 µg/kg for OTA and OTB. The LL was set at 6 µg/kg for AFB1 and the other detected mycotoxins [16].

(2) Mycotoxin frequency for each treatment (%) = (Number of positive samples/Total number of samples) × 100

Principal component analysis (PCA) was conducted in IBM SPSS Statistics 21. The data consisted of a binary matrix indicating whether each fungal isolate was present (1) or absent (0). The correlation matrix was used as the basis for PCA, extracting three principal components without rotation. Factor scores were generated with the regression method, stored as new variables, and then compared among treatments, application numbers, and storage times to produce score plots.

## Figures and Tables

**Table 1:** Mean values ± standard deviation (SD) (n = 60) of physicochemical quality parameters of dried figs subjected to preharvest applications of oxalic acid, γ-aminobutyric acid (GABA), and melatonin at commercial harvest.

		Moisture Content (%)			a_w_			Total Soluble Solids (TSS, °Brix)			Firmness (N)			Colour								
														L*			C*			Hue		
H1_2app *		** Mean **		**SD**	** Mean **		**SD**	** Mean **		**SD**	** Mean **		**SD**	** Mean **		**SD**	** Mean **		**SD**	** Mean **		**SD**
	Control	21.5	±	0.3 abc ^1^	0.54	±	0.00 c	81.6	±	0.1 ab	1.66	±	0.56	59.0	±	5.3	33.8	±	2.6	74.2	±	1.9
	C20	20.2	±	0.3 c	0.57	±	0.00 a	82.0	±	0.4 a	1.64	±	0.48	56.4	±	3.7	37.4	±	1.7	72.7	±	1.5
	A1	22.2	±	0.4 a	0.54	±	0.00 c	80.8	±	0.1 c	1.23	±	0.74	55.3	±	4.5	37.6	±	2.5	72.8	±	2.5
	A2	21.0	±	0.5 abc	0.53	±	0.00 d	81.7	±	0.1 a	1.36	±	0.52	56.9	±	6.8	37.6	±	2.0	73.0	±	3.3
	G10	20.3	±	0.2 c	0.53	±	0.00 e	81.7	±	0.2 a	1.29	±	0.53	58.7	±	4.4	36.7	±	2.8	73.8	±	2.5
	G50	22.1	±	0.6 ab	0.55	±	0.00 b	81.2	±	0.1 bc	1.54	±	0.46	52.5	±	7.0	34.6	±	4.9	72.9	±	2.8
	M0.1	20.9	±	0.3 bc	0.54	±	0.00 c	81.6	±	0.1 ab	1.60	±	0.64	50.8	±	8.9	34.4	±	3.3	72.2	±	3.0
	M0.5	20.4	±	0.5 c	0.54	±	0.00 cd	81.6	±	0.1 ab	1.24	±	0.29	56.1	±	10.3	34.7	±	4.4	74.1	±	3.3
** H2_2app **																						
	Control	28.0	±	0.7 ab	0.51	±	0.00 d	83.5	±	0.6 a	1.01	±	0.31	63.8	±	4.6	37.5	±	2.5	75.2	±	2.0
	C20	30.3	±	0.8 a	0.55	±	0.00 a	81.7	±	1.4 ab	0.66	±	0.21	59.5	±	6.4	35.6	±	3.4	74.0	±	2.4
	A1	26.9	±	0.9 ab	0.52	±	0.00 c	83.0	±	0.3 a	1.01	±	0.47	57.5	±	11.3	33.4	±	5.8	71.9	±	5.4
	A2	25.5	±	0.6 b	0.50	±	0.00 e	82.8	±	0.1 a	0.89	±	0.22	57.8	±	7.2	36.3	±	1.9	73.7	±	2.7
	G10	26.9	±	0.7 ab	0.54	±	0.00 b	82.0	±	0.4 ab	0.94	±	0.34	58.9	±	6.7	37.8	±	2.5	74.2	±	3.0
	G50	26.9	±	1.7 ab	0.52	±	0.00 c	82.5	±	0.6 ab	0.75	±	0.23	54.7	±	8.6	34.1	±	5.7	71.9	±	3.6
	M0.1	24.7	±	1.4 b	0.50	±	0.00 e	84.0	±	1.2 a	0.92	±	0.28	61.5	±	6.1	37.1	±	2.3	74.6	±	2.3
	M0.5	27.7	±	2.6 ab	0.55	±	0.00 a	79.9	±	1.4 b	0.75	±	0.16	59.8	±	5.7	36.5	±	1.6	74.1	±	2.0
** H1_3app **																						
	Control	28.0	±	0.7	0.51	±	0.00 f	83.5	±	0.6 a	1.01	±	0.31	63.8	±	4.6	37.5	±	2.5	75.2	±	2.0
	C20	26.6	±	0.4	0.51	±	0.00 g	83.4	±	1.2 a	0.90	±	0.26	57.4	±	7.2	36.4	±	5.6	73.3	±	3.9
	A1	27.5	±	0.4	0.54	±	0.00 c	81.5	±	0.5 ab	0.83	±	0.28	61.3	±	5.6	37.9	±	2.4	75.0	±	2.0
	A2	26.7	±	0.4	0.55	±	0.00 b	81.7	±	0.4 ab	0.90	±	0.24	57.7	±	7.2	36.4	±	3.7	73.0	±	3.2
	G10	27.3	±	1.7	0.57	±	0.00 a	79.2	±	0.1 b	0.83	±	0.20	61.0	±	6.5	39.1	±	1.7	74.6	±	2.5
	G50	27.1	±	1.4	0.52	±	0.00 e	82.4	±	0.4 a	0.77	±	0.17	61.7	±	8.8	36.3	±	2.7	75.5	±	2.9
	M0.1	27.2	±	0.2	0.55	±	0.00 bc	83.1	±	1.6 a	0.91	±	0.31	60.6	±	6.1	39.0	±	2.2	73.8	±	2.2
	M0.5	26.2	±	0.7	0.54	±	0.00 d	82.6	±	0.4 a	0.94	±	0.17	60.5	±	6.6	37.1	±	2.7	74.4	±	2.6
** H2_3app **																						
	Control	25.0	±	0.6 a	0.51	±	0.00 a	81.9	±	0.4 c	1.35	±	0.38	57.5	±	6.7 ab	35.3	±	2.3 b	74.0	±	2.5
	C20	24.5	±	1.6 a	0.51	±	0.00 b	83.2	±	0.3 b	1.59	±	0.36	57.7	±	5.5 ab	38.1	±	1.8 ab	73.9	±	2.0
	A1	22.8	±	0.7 ab	0.49	±	0.00 d	83.3	±	0.4 b	1.57	±	0.62	57.7	±	7.4 ab	38.8	±	3.9 ab	73.3	±	2.6
	A2	22.4	±	0.4 ab	0.45	±	0.00 g	84.9	±	0.1 a	2.12	±	0.37	52.2	±	8.4 b	35.4	±	4.1 b	71.4	±	3.0
	G10	22.3	±	0.4 ab	0.47	±	0.00 e	84.3	±	0.3 ab	1.84	±	0.45	56.2	±	7.6 ab	35.9	±	2.6 ab	73.0	±	2.4
	G50	20.3	±	2.5 b	0.47	±	0.00 e	85.0	±	0.0 a	2.14	±	1.05	63.2	±	3.5 a	39.7	±	1.3 a	74.9	±	1.5
	M0.1	22.3	±	0.2 ab	0.46	±	0.00 f	84.0	±	0.6 ab	2.19	±	0.75	63.2	±	3.5 a	39.7	±	1.3 a	74.9	±	1.5
	M0.5	23.3	±	1.1 ab	0.49	±	0.00 c	81.9	±	0.4 c	1.79	±	0.47	57.1	±	6.0 ab	37.5	±	2.8 ab	73.3	±	2.5
** Total Harvest **																						
	H1_2app	21.0	±	0.8 C ^2^	0.54	±	0.01	81.5	±	0.4 C	1.45	±	0.54B	55.7	±	7.0 B	35.8	±	3.4 B	73.2	±	2.6
	H2_2app	27.1	±	2.0 A	0.53	±	0.02	82.4	±	1.4 B	0.87	±	0.30C	59.2	±	7.5 A	36.0	±	3.7 B	73.7	±	3.2
	H1_3app	27.1	±	0.9 A	0.54	±	0.02	82.2	±	1.5 B	0.89	±	0.25C	60.5	±	6.7 A	37.5	±	3.2 A	74.4	±	2.7
	H2_3app	23.0	±	1.6 B	0.48	±	0.02	83.6	±	1.2 A	1.82	±	0.64A	58.1	±	6.9 AB	37.6	±	3.1 A	73.6	±	2.4
*** p *** ** -treatment **		<0.05			<0.05			<0.05			>0.05			>0.05			>0.05			>0.05		
*** p *** ** -applications **		<0.05			>0.05			<0.05			<0.05			<0.05			<0.05			>0.05		
*** p *** ** -treatment x ** ** applications **		<0.05			<0.05			<0.05			<0.05			<0.05			<0.05			>0.05		

^1^ In each column, lowercase letters show significant differences among treatments (*p* < 0.05). ^2^ In each column, capital letters show significant differences among harvest times (*p* < 0.05). * H1_2app: First harvest with two applications; H2_2app: Second harvest with two applications; H1_3app: First harvest with three applications; H2_3app: Second harvest with three applications; Control: untreated; C20: treated with 0.2 mL/L Tween 20; A1: treated with 1 mM OA; A2: treated with 2 mM OA; G10: treated with 10 mM GABA; G50: treated with 50 mM GABA; M0.1: treated with 0.1 mM MT; M0.5: treated with 0.5 mM MT.

**Table 2:** Mycotoxin profiles (expressed as % of samples) of dried figs treated with preharvest elicitors after six months of storage.

Mycotoxin	Range	Control ^4^	C20	A1	A2	G10	G50	M0.1	M0.5
OTA ^3^	<LOD	66.7	50.0	75.0	75.0	75.0	75.0	75.0	100
	LOD-LL ^1^	33.3	50.0	25.0	25.0	25.0	25.0	25.0	-
	>LL	-	-	-	-	-	-	-	-
OTB	<LOD	100	100	100	100	100	75.0	100	100
	LOD-LL ^1^	-	-	-	-	-	25.0	-	-
	>LL	-	-	-	-	-	-	-	-
AFB_1_	<LOD	100	100	100	75.0	100	100	100	100
	LOD-LL ^2^	-	-	-	25.0	-	-	-	-
	>LL	-	-	-	-	-	-	-	-
AFB_2_	<LOD	100	100	100	75.0	100	100	100	100
	LOD-LL ^2^	-	-	-	-	-	-	-	-
	>LL	-	-	-	25.0	-	-	-	-
CIT	<LOD	100	100	100	75.0	100	100	100	100
	LOD-LL ^2^	-	-	-	-	-	-	-	-
	>LL	-	-	-	25.0	-	-	-	-
AOH	<LOD	100	100	75.0	100	100	100	100	100
	LOD-LL ^2^	-	-	25.0	-	-	-	-	-
	>LL	-	-	-	-	-	-	-	-

^1^ LL = 8 µg/kg; ^2^ LL = 6 µg/kg. ^3^ OTA: ochratoxin A; OTB: ochratoxin B; AFB_1_: aflatoxin B_1_; AFB_2_: aflatoxin B_2_; CIT: citrinin; AOH: alternariol. ^4^ Control: untreated; C20: treated with 0.2 mL/L Tween 20; A1: treated with 1 mM OA; A2: treated with 2 mM OA; G10: treated with 10 mM GABA; G50: treated with 50 mM GABA; M0.1: treated with 0.1 mM MT; M0.5: treated with 0.5 mM MT.

**Table 3:** Sampling schedule at commercial harvest and during storage.

Harvest Time	Applications	Code	Sampling Date		
			Commercial Harvest	3 Months	6 Months
H1	2app	H1_2app *	7 August 2023	7 November 2023	7 February 2024
H2		H2_2app	14 August 2023	14 November 2023	14 February 2024
H1	3app	H1_3app	14 August 2023	14 November 2023	14 February 2024
H2		H2_3app	21 August 2023	21 November 2023	21 February 2024

* H1_2app: First harvest, two applications; H2_2app: Second harvest, two applications; H1_3app: First harvest, three applications; H2_3app: Second harvest, three applications.

## Data Availability

The original contributions presented in this study are included in the article/Supplementary Material. Further inquiries can be directed to the corresponding author.

## Supplementary Materials

The following supporting information can be downloaded at: https://www.mdpi.com/article/10.3390/toxins18030140/s1, Table S1: Mean values ± SD (*n* = 3) of microbiological quality parameters of dried figs at commercial harvest. Table S2: Mean values ± SD (*n* = 60) of physicochemical quality parameters of dried figs during storage. Table S3: Mean values ± SD (*n* = 3) of microbiological quality parameters (log CFU/g) of dried figs during storage. Table S4: Accession numbers of DNA sequences used for fungal identification. Table S5: Retention time (RT), limit of detection (LOD), limit of quantification (LOQ), and commercial source of mycotoxin standard analysed. Figure S1: Daily maximum, mean, and minimum temperature (°C) and relative humidity (%) at Finca La Orden (Guadajira, Spain) during the experimental period (July–August 2023) (REDAREX, 2023).

## Abbreviations

The following abbreviations are used in this manuscript:

GABA	γ -aminobutyric acid
MT	Melatonin
OA	Oxalic acid
H1_2app	First harvest with two applications
H2_2app	Second harvest with two applications
H1_3app	First harvest with three applications
H2_3app	Second harvest with three applications
C20	Treated with 0.2 mL/L Tween 20
A1	Treated with 1 mM OA
A2	Treated with 2 mM OA
G10	Treated with 10 mM GABA
G50	Treated with 50 mM GABA
M0.1	Treated with 0.1 mM MT
M0.5	Treated with 0.5 mM MT
OTA	Ochratoxin A
OTB	Ochratoxin B
AFs	Aflatoxins
AFB_1_	Aflatoxin B_1_
AFB_2_	Aflatoxin B_2_
AFG_1_	Aflatoxin G_1_
AFG_2_	Aflatoxin G_2_
CIT	Citrinin
AOH	Alternariol
FB_1_	Fumonisin B_1_
CPA	α-cyclopiazonic acid
Myc Ac	Mycophenolic acid
AME	Alternariol monomethyl ether
TeA	Tenuazonic acid
ZEA	Zearalenone
STG	Sterigmatocystin
OM-STG	*O*-methylsterigmatocystin
GRIS	Griseofulvin
<LOD	Below the limit of detection
LOD-LL	Between limit of detection and the legal limit
>LL	Above the legal limit
a_w_	Water activity
TSS	Total soluble solids
L*	Brightness
C*	Chroma
PC	Principal component
PCA	Principal component analysis
