# Supplementary material for: Effects of Preharvest Application of Oxalic Acid, γ-Aminobutyric Acid, and Melatonin on the Microbiological and Physicochemical Quality of Dried Figs at Commercial Harvest and During Storage
Source: Toxins (Basel). 2026 Mar 13;18(3):140. doi: 10.3390/toxins18030140 (PMC13030335; doi:10.3390/toxins18030140)
Supplement: Supplementary file 1 [file toxins-18-00140-s001.zip › toxins-4170456-supplementary.pdf]

Supplementary Material

Effects of Preharvest Application of Oxalic Acid, γ-Aminobutyric Acid, and Melatonin on the Microbiological and Physicochemical Quality of Dried Figs at Commercial Harvest and During Storage

Cristina Hidalgo [1,2], Santiago Ruiz-Moyano [1,2], Alicia Rodríguez [1,2,*], María G. Cordoba [1,2], Margarita López-Corrales [3] and Manuel J. Serradilla [4]

[1] Nutrición y Bromatología, Escuela de Ingenierías Agrarias, Universidad de Extremadura,
06007 Badajoz, Spain; cristinahr@unex.es (C.H.); srmsh@unex.es (S.R.-M.); mdeguia@unex.es (M.G.C.)

[2] Instituto Universitario de Investigación en Recursos Agrarios (INURA), Avd. de la Investigación,
Universidad de Extremadura, 06006 Badajoz, Spain

[3] Junta de Extremadura, Centro de Investigación Finca La Orden-Valdesequera (CICYTEX), Fruticultura, Autovía Madrid-Lisboa, s/n, 06187 Guadajira, Spain; margarita.lopez@juntaex.es

[4] Área de Postcosecha, Valorización Vegetal y Nuevas Tecnologías, Centro de Investigaciones Científicas y Tecnológicas de Extremadura (CICYTEX), Instituto Tecnológico Agroalimentario de Extremadura
(INTAEX), Junta de Extremadura, Avda. Adolfo Suárez, s/n, 06007 Badajoz, Spain;
manuel.serradilla@juntaex.es

* Correspondence: aliciarj@unex.es; Tel.: +34-924489283

This supplementary material contains 1 figure and 5 tables.

Table S1. Mean values ± SD (n = 3) of microbiological quality parameters of dried figs at commercial harvest.

	Mould Counts (log CFU/g)			Yeast Counts (log CFU/g)		
**H1_2app***						
Control	3.31	±	0.80	0.00	±	0.00
C20	2.39	±	0.36	0.00	±	0.00
A1	3.09	±	0.43	1.41	±	2.44
A2	2.58	±	0.17	0.77	±	1.33
G10	2.83	±	0.62	1.79	±	1.70
G50	2.64	±	0.73	0.87	±	1.50
M0.1	3.73	±	0.98	0.00	±	0.00
M0.5	3.38	±	0.11	0.00	±	0.00
**H2_2app**						
Control	2.73	±	0.05	0.77	±	1.33
C20	0.83	±	1.43	4.97	±	1.81
A1	4.17	±	1.27	0.00	±	0.00
A2	2.11	±	1.95	2.21	±	3.82
G10	3.73	±	0.74	3.62	±	3.18
G50	3.83	±	1.29	0.00	±	0.00
M0.1	2.80	±	0.08	0.77	±	1.33
M0.5	2.67	±	0.91	0.00	±	0.00
**H1_3app**						
Control	2.73	±	0.05	0.77	±	1.33
C20	3.11	±	1.68	1.84	±	3.18
A1	2.40	±	0.46	0.00	±	0.00
A2	2.30	±	0.30	3.27	±	0.91
G10	3.74	±	3.33	2.15	±	3.73
G50	3.91	±	1.55	1.33	±	1.15
M0.1	2.24	±	2.21	3.49	±	1.50
M0.5	3.69	±	0.27	0.00	±	0.00
**H2_3app**						
Control	3.75	±	1.66	2.76	±	3.20
C20	3.15	±	0.81	0.67	±	1.15
A1	2.64	±	0.30	0.00	±	0.00
A2	4.02	±	1.68	0.83	±	1.43
G10	3.72	±	0.32	0.00	±	0.00
G50	2.87	±	0.47	0.00	±	0.00
M0.1	2.97	±	0.25	1.33	±	1.15
M0.5	3.59	±	0.47	0.00	±	0.00
**Total Harvest**						
**H1_2app**	2.99	±	0.67	0.60	±	1.26
**H2_2app**	2.86	±	1.41	1.54	±	2.45
**H1_3app**	3.02	±	1.52	1.61	±	2.07
**H2_3app**	3.34	±	0.91	0.70	±	1.47
***p*-treatment**		>0.05			>0.05	
***p*-applications**		>0.05			>0.05	
***p*- treatment x applications**		>0.05			>0.05	

*H1_2app: First harvest with two applications; H2_2app: Second harvest with two applications; H1_3app: First harvest with three applications; H2_3app: Second harvest with three applications; Control: untreated; C20: treated with 0.2 mL/L Tween 20; A1: treated with 1 mM OA; A2: treated with 2 mM OA; G10: treated with 10 mM GABA; G50: treated with 50 mM GABA; M0.1: treated with 0.1 mM MT; M0.5: treated with 0.5 mM MT.

Table S2. Mean values ± SD (n = 60) of physicochemical quality parameters of dried figs during storage.

	Moisture content (%)	a_w	Total Soluble Solids (TSS, ºBrix)	Firmness (N)	Colour		
					L*	C*	hue
**Control***							
**Harvest**	24.8 ± 2.9	0.52 ± 0.02	82.3 ± 1.0a	1.34 ± 0.50	60.1 ± 6.1	35.6 ± 2.8	74.5 ± 2.2
**3 months**	43.0 ± 3.2	0.70 ± 0.01	68.5 ± 0.9b	0.57 ± 0.25	60.0 ± 6.1	34.0 ± 2.2	76.3 ± 3.1
**6 months**	40.6 ± 3.3	0.71 ± 0.00	66.3 ± 0.5c	0.55 ± 0.20	57.9 ± 5.5	32.5 ± 3.3	76.4 ± 2.6
**C20**							
**Harvest**	25.4 ± 3.9	0.53 ± 0.03	82.6 ± 1.1a	1.20 ± 0.55	57.7 ± 5.7	36.9 ± 3.5	73.5 ± 2.5
**3 months**	43.3 ± 4.8	0.70 ± 0.01	68.4 ± 0.5b	0.47 ± 0.17	58.8 ± 7.5	34.6 ± 2.8	76.4 ± 3.9
**6 months**	41.9 ± 3.7	0.71 ± 0.01	66.1 ± 0.6c	0.45 ± 0.20	56.0 ± 8.5	32.8 ± 3.9	75.3 ± 4.5
**A1**							
**Harvest**	24.8 ± 2.5	0.52 ± 0.03	82.1 ± 1.1a	1.16 ± 0.60	57.9 ± 7.7	36.9 ± 4.3	73.3 ± 3.4
**3 months**	41.8 ± 3.5	0.69 ± 0.01	69.6 ± 0.7b	0.50 ± 0.19	57.1 ± 9.0	32.5 ± 5.1	76.6 ± 3.6
**6 months**	39.2 ± 2.6	0.70 ± 0.01	66.7 ± 0.3c	0.48 ± 0.19	56.1 ± 7.4	32.7 ± 4.1	76.2 ± 3.4
**A2**							
**Harvest**	23.9 ± 2.4	0.51 ± 0.04	82.8 ± 1.4a	1.32 ± 0.61	56.1 ± 7.5	36.4 ± 3.1	72.8 ± 3.1
**3 months**	42.0 ± 3.7	0.70 ± 0.00	69.3 ± 0.5b	0.46 ± 0.20	58.7 ± 7.2	34.5 ± 4.4	76.2 ± 4.0
**6 months**	40.8 ± 2.4	0.70 ± 0.01	67.3 ± 1.2c	0.50 ± 0.19	57.5 ± 7.5	33.4 ± 3.4	76.0 ± 3.2
**G10**							
**Harvest**	24.2 ± 3.2	0.53 ± 0.04	81.8 ± 1.9a	1.23 ± 0.55	58.7 ± 6.4	37.4 ± 2.6	73.9 ± 2.6
**3 months**	43.9 ± 2.6	0.70 ± 0.02	68.4 ± 1.1b	0.52 ± 0.20	58.7 ± 5.3	34.7 ± 2.1	76.2 ± 3.5
**6 months**	42.1 ± 4.3	0.71 ± 0.01	66.3 ± 0.9c	0.48 ± 0.19	56.5 ± 8.6	32.1 ± 4.5	76.1 ± 4.3
**G50**							
**Harvest**	23.7 ± 3.2	0.51 ± 0.03	82.8 ± 1.5a	1.30 ± 0.82	58.0 ± 8.3	36.2 ± 4.5	73.8 ± 3.1
**3 months**	42.9 ± 3.8	0.70 ± 0.01	68.1 ± 0.8b	0.49 ± 0.19	58.2 ± 9.3	33.9 ± 4.7	76.4 ± 4.7
**6 months**	41.2 ± 4.6	0.70 ± 0.01	66.6 ± 1.0c	0.49 ± 0.22	57.9 ± 11.1	32.0 ± 5.2	76.9 ± 5.0
**M0.1**							
**Harvest**	23.8 ± 2.6	0.51 ± 0.04	83.2 ± 1.4a	1.41 ± 0.75	59.0 ± 7.9	37.6 ± 3.1	73.9 ± 2.5

**3 months**	42.8 ± 2.0	0.70 ± 0.01	68.0 ± 0.6b	0.50 ± 0.21	56.6 ± 7.7	33.4 ± 3.6	75.6 ± 4.3
**6 months**	40.6 ± 3.1	0.71 ± 0.01	66.3 ± 1.5c	0.50 ± 0.20	57.0 ± 7.9	32.6 ± 5.0	75.9 ± 4.2
**M0.5**							
**Harvest**	24.2 ± 3.2	0.53 ± 0.03	81.5 ± 1.2a	1.18 ± 0.49	58.4 ± 7.3	36.4 ± 3.1	73.9 ± 2.6
**3 months**	39.5 ± 4.0	0.70 ± 0.01	68.4 ± 1.0b	0.48 ± 0.18	59.8 ± 6.3	34.6 ± 2.7	77.5 ± 3.6
**6 months**	39.4 ± 3.5	0.71 ± 0.01	66.2 ± 0.8c	0.47 ± 0.19	58.3 ± 9.7	31.9 ± 5.0	77.1 ± 4.4
**Total Storage**							
**Harvest**	24.3 ± 3.0B	0.52 ± 0.03C	82.4 ± 1.4A	1.26 ± 0.62A	58.2 ± 7.2	36.7 ± 3.5A	73.7 ± 2.8B
**3 months**	42.3 ± 2.1A	0.70 ± 0.01B	68.6 ± 0.9B	0.50 ± 0.20B	58.4 ± 7.4	34.0 ± 3.7B	76.4 ± 3.9A
**6 months**	40.7 ± 3.5A	0.70 ± 0.01A	66.5 ± 1.0C	0.49 ± 0.20B	57.1 ± 8.4	32.5 ± 4.4C	76.2 ± 4.1A
***p*-treatment**	>0.05	>0.05	<0.05	>0.05	>0.05	>0.05	>0.05
***p*-storage**	<0.05	<0.05	<0.05	<0.05	<0.05	<0.05	<0.05
***p*-treatment x storage**	>0.05	>0.05	<0.05	>0.05	>0.05	>0.05	>0.05

[1]In each column, lowercase letters indicate significant differences among treatments ($p < 0.05$).

[2] In each column, capital letters indicate significant differences among storage times ($p < 0.05$).

*Control: untreated; C20: treated with 0.2 mL/L Tween 20; A1: treated with 1 mM OA; A2: treated with 2 mM OA; G10: treated with 10 mM GABA; G50: treated with 50 mM GABA; M0.1: treated with 0.1 mM MT; M0.5: treated with 0.5 mM MT.

Table S3. Mean values ± SD (n = 3) of microbiological quality parameters (log CFU/g) of dried figs during storage.

	Mould Counts (log CFU/g)			Yeast Counts (log CFU/g)		
**Control***						
**Harvest**	3.26	±	1.02	1.17	±	2.13
**3 months**	3.16	±	1.16	2.39	±	2.20
**6 months**	2.76	±	0.39	0.97	±	1.51
**C20**						
**Harvest**	2.37	±	1.41	1.87	±	2.58
**3 months**	3.21	±	0.70	2.10	±	1.92
**6 months**	3.14	±	0.79	0.83	±	1.34
**A1**						
**Harvest**	3.07	±	0.94	0.35	±	1.22
**3 months**	3.07	±	0.91	0.69	±	1.02
**6 months**	3.05	±	0.62	1.50	±	1.76
**A2**						
**Harvest**	2.75	±	1.36	1.77	±	2.16
**3 months**	2.66	±	0.96	1.05	±	1.36
**6 months**	3.09	±	0.81	0.76	±	1.13
**G10**						
**Harvest**	3.51	±	1.54	1.89	±	2.59
**3 months**	3.49	±	1.08	0.53	±	0.95
**6 months**	3.28	±	1.27	0.41	±	0.96
**G50**						
**Harvest**	3.31	±	1.11	0.55	±	1.01
**3 months**	3.15	±	0.76	0.73	±	1.36
**6 months**	2.98	±	0.56	0.58	±	1.08
**M0.1**						
**Harvest**	2.93	±	1.18	1.40	±	1.68
**3 months**	2.64	±	0.79	0.86	±	1.06
**6 months**	2.47	±	0.82	1.15	±	1.21
**M0.5**						
**Harvest**	3.33	±	0.62	0.00	±	0.00
**3 months**	3.49	±	0.77	1.36	±	1.80
**6 months**	3.30	±	0.70	1.67	±	1.93
**Total Storage**						
**Harvest**	3.06	±	1.19	1.12	±	1.92
**3 months**	3.11	±	0.91	1.18	±	1.57
**6 months**	3.02	±	0.80	0.99	±	1.41
***p*-treatment**		<0.05			>0.05	
***p*-storage**		>0.05			>0.05	
***p*-treatment x storage**		>0.05			<0.05	

*Control: untreated; C20: treated with 0.2 mL/L Tween 20; A1: treated with 1 mM OA; A2: treated with 2 mM OA; G10: treated with 10 mM GABA; G50: treated with 50 mM GABA; M0.1: treated with 0.1 mM MT; M0.5: treated with 0.5 mM MT.

Table S4. Accession numbers of DNA sequences used for fungal identification.

Fungal Identification	GenBank Accession Number			
	**β-tubulin**	***CaM***	***gpd***	**ITS**
*Aspergillus welwitschiae**	MT410111.1	-	-	-
*Aspergillus niger**	ON803641.1	-	-	-
Aspergillus tubingensis	KY990200.1	PV563440.1	-	-
Aspergillus flavus	JX545049.1	HF570047.1	-	-
Aspergillus minisclerotigenes	MK119727.1	JX456196.1	-	-
Aspergillus europaeus	LN909006.1	LT899571.1	-	-
Aspergillus uvarum	HE984421.1	HE984437.1	-	-
Aspergillus brasiliensis	AM295185.1	AM295176.1	-	-
Aspergillus terreus	JX501418.1	MK451535.1	-	-
Aspergillus melleus	-	EF661391.1	-	-
Aspergillus ochraceus	-	LR693990.1	-	-
Aspergillus sydowii	OM960826.1	LN898795.1	-	-
Aspergillus rugulosus	AB524361.1	EF591687.1	-	-
Aspergillus alliaceus	MG517616.1	MG518161.1	-	-
Aspergillus neoalliaceus	MG517613.1	MG518161.1	-	-
Alternaria alternata	-	-	MZ835392.1	OQ456129.1
Alternaria botrytis	MF175214.1	-	AY278817.1	
Alternaria tenuissima	-	-	OR603956.1	KT384235.1
Alternaria terricola	-	-	FJ266501.1	OR147949.1
Alternaria consortialis	-	-	MW658282.1	OR143736.1
Alternaria cucurbitae	KU324180.1	-	KU324180.1	
Alternaria arborescens	-	-	PV130457.1	OK447899.1
Alternaria atra	-	-	PQ352303.1	
Alternaria gaisen	-	-	OL754635.1	
Alternaria infectoria	MK567924.1	-	FJ214811.1	OP896222.1
Cladosporium cladosporioides	EF101453.1	-	-	MK722298.1
Cladosporium herbarum	-	-	-	MH863981.1
Cladosporium oxysporum	PP907108.1	-	-	OQ608652.1
Cladosporium anthropophilum	-	-	-	MF472927.1
Cladosporium angustiporum	-	-	-	MH863862.1
Cladosporium perangustum	-	-	-	LN834380.1
Cladosporium subuliforme	-	-	-	MH864124.1
Penicillium citrinum	LT559007.1	-	-	ON127876.1
Penicillium oxalicum	OP220035.1	-	-	MK163534.1
Penicillium sumatraense	LT559016.1	-	-	MH864543.1
Penicillium georgiense	EF506224.1	-	-	OW987618.1
Penicillium paneum	KJ866977.1	-	-	MH863983.1
Penicillium brevicompactum	AF125943.1	-	-	AF125943.1
Penicillium chrysogenum	AY495981.1	-	-	MH865449.1
Fusarium verticillioides	MT011054.1	-	-	PQ657487.1
Fusarium solani	OP375799.1	-	-	ON003463.1
Fusarium proliferatum	KU603920.1	-	-	LT970807.1
Fusarium fujikuroi	MN896949.1	-	-	OM995870.1
Fusarium equiseti	OP680529.1	-	-	OQ421751.1
Talaromyces amestolkiae				NR_120179.1
Talaromyces adpressus	MH792911.1			
Talaromyces radicus	JX494299.1			MH862702.1

Talaromyces variabilis	KF984643.1	PP860283.1
Talaromyces rugulosus	MW162413.1	OR237670.1
Trichoderma gamsii		MZ695283.1
Epicoccum nigrum	OL870947.1	AJ279448.1
Truncatella angustata	MH554696.1	MH860182.1
Albifimbria verrucaria	KU845965.1	KU845890.1
Albifimbria viridis	KU845975.1	KU845899.1
Boeremia exigua	MW273782.1	EU167567.1
Botryosphaeria fabicerciana	OP758199.1	KU866657.1
Biscogniauxia mediterranea	KM267203.1	MT819840.1
Stemphylium vesicarium	MN410922.1	MW245002.1
Stemphylium eturmiunum	LC601946.1	MW940706.1
Paecilomyces formosus	MK330005.1	MZ021409.1
Pseudopithomyces chartarum		MH860227.1

Fungal identification was performed using a polyphasic approach as described in section 5.2.3. Partial *β-tubulin* (Bt) gene sequences were obtained for all isolates. *Aspergillus niger* and *A. welwitschiae* (*) were differentiated by specific nucleotide positions in the Bt gene, with calmodulin (*CaM*) sequencing used for further confirmation of specific genotypes. Other Aspergillus species were confirmed by *CaM* sequencing. Alternaria species were identified using the glyceraldehyde-3-phosphate dehydrogenase (*gpd*) gene. Other fungal genera (e.g., *Cladosporium* spp., *Penicillium* spp., *Fusarium* spp.) were confirmed using the internal transcribed spacer (*ITS*) region

Table S5. Retention time (RT), limit of detection (LOD), limit of quantification (LOQ), and commercial source of mycotoxin standard analysed.

Mycotoxins	RT (min)	LOD (ng/mL)	LOQ (ng/mL)	Commercial Source
Aflatoxin G_1 (AFG_1)	1.41±0.02	0.17	0.5	LGC Standard (Middlesex, UK)
Aflatoxin G_2 (AFG_2)	1.24±0.02	0.17	0.5	LGC Standard
Aflatoxin B_1 (AFB_1)	1.68±0.02	0.17	0.5	LGC Standard
Aflatoxin B_2 (AFB_2)	1.48±0.02	0.17	0.5	LGC Standard
Ochratoxin A (OTA)	3.34±0.02	0.33	1	Merck (Darmstadt, Germany)
Ochratoxin B (OTB)	2.45±0.02	0.17	0.5	Merck
Citrinin (CIT)	2.40±0.02	0.17	0.5	Merck
α-Cyclopiazonic acid (CPA)	4.24±0.02	0.33	1	Merck
Mycophenolic Acid (Myc Ac)	2.34±0.02	0.33	1	Merck
Zearalenone (ZEA)	3.39±0.02	0.17	0.5	Merck
O-Methylsterigmatocystin (OM-STG)	2.84±0.02	0.17	0.5	Santa Cruz Biotechnology (Heidelberg, Germany)
Sterigmatocystin (STG)	3.86±0.02	0.17	0.5	Sigma
Fumonisin B_1 (FB_1)	1.18±0.02	0.66	2	Thermo Scientific Chemicals (Waltham, USA)
Griseofulvin (GRIS)	2.51±0.02	0.17	0.5	Focus Biomolecules (Plymouth Meeting, USA)
Alternariol (AOH)	3.45±0.02	0.17	0.5	Cayman Chemical (Ann Arbor, USA)
Alternariol-monomethyl ether (AME)	1.85±0.02	0.17	0.5	Cayman Chemical

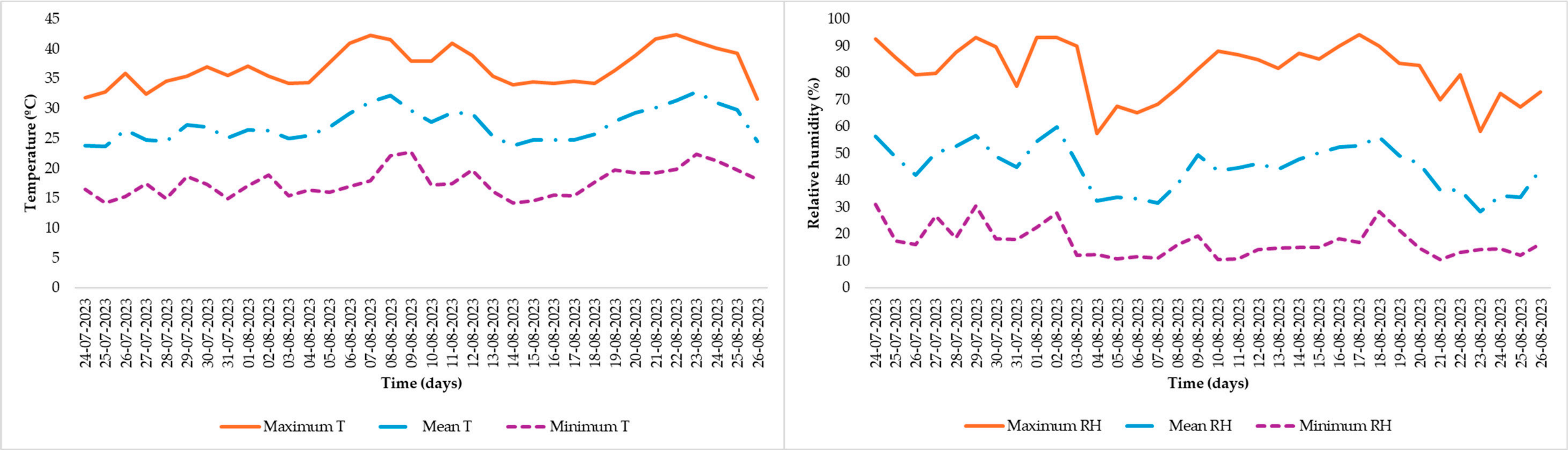

Figure S1. Daily maximum, mean, and minimum temperature (°C) and relative humidity (%) at Finca La Orden (Guadajira, Spain) during the experimental period (July–August 2023). (REDAREX, 2023).
